# An Updated Review on Prebiotics: Insights on Potentials of Food Seeds Waste as Source of Potential Prebiotics

**DOI:** 10.3390/molecules27185947

**Published:** 2022-09-13

**Authors:** Gafar Babatunde Bamigbade, Athira Jayasree Subhash, Afaf Kamal-Eldin, Laura Nyström, Mutamed Ayyash

**Affiliations:** 1 Department of Food Science, College of Agriculture and Veterinary Medicine, United Arab Emirates University (UAEU), Al-Ain P.O. Box 15551, United Arab Emirates; 2 Department of Health Science and Technology, Institute of Food, Nutrition and Health, ETH Zurich, 8092 Zurich, Switzerland

**Keywords:** fructo-oligosaccharides, galactooligosaccharides, prebiotics, dietary fiber, gastrointestinal tract

## Abstract

Prebiotics are a group of biological nutrients that are capable of being degraded by microflora in the gastrointestinal tract (GIT), primarily Lactobacilli and Bifidobacteria. When prebiotics are ingested, either as a food additive or as a supplement, the colonic microflora degrade them, producing short-chain fatty acids (SCFA), which are simultaneously released in the colon and absorbed into the blood circulatory system. The two major groups of prebiotics that have been extensively studied in relation to human health are fructo-oligosaccharides (FOS) and galactooligosaccharides (GOS). The candidature of a compound to be regarded as a prebiotic is a function of how much of dietary fiber it contains. The seeds of fruits such as date palms have been reported to contain dietary fiber. An increasing awareness of the consumption of fruits and seeds as part of the daily diet, as well as poor storage systems for seeds, have generated an enormous amount of seed waste, which is traditionally discarded in landfills or incinerated. This cultural practice is hazardous to the environment because seed waste is rich in organic compounds that can produce hazardous gases. Therefore, this review discusses the potential use of seed wastes in prebiotic production, consequently reducing the environmental hazards posed by these wastes.

## 1. Background

Due to poor nutrition, increases in food-borne illnesses, and tobacco and alcohol consumption, there is a high rate of morbidity and mortality worldwide. Non-communicable diseases, such as chronic obesity, gastrointestinal disorders, diabetes, coronary heart diseases, cancer, and degenerative diseases are on the increase; hence, the situation has led to an increased interest in prebiotics in recent years. Prebiotics have been described by Patel and Goyal as undigestible food ingredients, such as polysaccharide and non-digestible oligosaccharides (NDO), that can selectively enhance the growth of beneficial microbes (lactic acid bacteria (LAB)) and exhibit antagonistic activity against pathogenic microorganisms in the gastro-intestinal tract (GIT), such as *Salmonella* sp. and *Escherichia coli*, to reduce their survival in the gut, thereby improving the host’s healthPatel and Goyal [1]. Gibson et al. [2] described the key characteristics of prebiotics and included fermentation by intestinal microflora, hydrolysis by mammalian and bacterial enzymes and gastrointestinal absorption, resistance to gastric acidity, ability to withstand food treatment processes, and selective stimulation of the growth and activity of intestinal bacteria, such as Lactobacilli or Bifidobacteria.

Variations exist in the types of known prebiotics, based on their source and chemical characteristics. Several studies have classified inulin, fructo-oligosaccharides (FOS), galactooligosaccharides (GOS), lactulose, and polydextose as standard prebiotics, while isomaltooligosaccharides (IMO), xylooligosaccahrides (XOS), and lactitol were categorized as emerging prebiotics by Sabater-Molina et al. [3,4], Femia et al. [5], and Figueroa-González et al. [6]. Mannitol, maltodextrin, raffinose, lactulose, and sorbitol are also prebiotics with proven health properties [7,8,9]. Vaidya and Sheth [10] considered resistant starch-rich whole grains as prebiotics due to the assumption of numerous advantages that could be derived from including them in meals. However, the absorption of these substances in the small intestine of healthy persons is not possible; rather, they undergo fermentation by the native flora of the colon, producing short-chain fatty acids (SCFAs). The fermentation ability of dietary fibers, such as oat β-glucan, xylans, hemicellulose, and cellulose to produce SCFAs reveal their potential to be regarded as prebiotics for humans [11,12].

Globally, there exist differences in the regulatory context of prebiotics [13]. The regulation of other functional foods (prebiotic-inclusive) was first documented in Japan with the implementation of the Foods for Specified Health Use (FOSHU) Act in the 1980s [13]. All foods and beverages known to have health benefits are certified by the Japanese Ministry of Health, Labor, and Welfare once the claims have been scientifically established. These foods are then allowed to be commercially marketed in Japan [13]. However, in Europe, the regulation of functional foods was first actioned in Sweden, then in the Netherlands and the United Kingdom, and later came together to form the Joint Health Initiative (JHCI) [13]. In 2002, the European Food Safety Authority (EFSA) was established to regulate the direct and indirect impacts of food safety. Since its inception, the EFSA has only certified one prebiotic, chicory inulin, giving it an EU health claim of contributing to the maintenance of normal defecation by increasing stool frequency [14]. It is expected that the daily consumption of 12 g of native chicory inulin should be taken for the claimed effect to be obtained [13]. The Food and Drug Administration was established in the United States in 1990 to assess the health claims made for food supplements [13]. However, prebiotics are yet to be recognized by the FDA and the purported health claims of microbiota modification are not yet accepted as a verified health claim [13]. Similarly, in some developing Asian countries, there are no official regulations for prebiotics and other functional foods [13].

Although dietary fibers are non-digestible polysaccharides, not all dietary fibers can be regarded as prebiotics; hence, not all non-digestible polysaccharides are prebiotics. The scientific conference called by the Food and Agriculture Organization of the United Nations (FAO) to discuss the positive impact of prebiotics on food was reviewed in the study by Pineiro et al. [15], who reported that the establishment of guidelines, criteria recommendations and the formulation of an analytical procedure for evaluating and establishing the non-hazardous use of prebiotics was the outcome reported by the international team that attended the conference.

A great deal of solid waste is generated from fruit processing [16]. This waste becomes an environmental hazard due to deposition in rivers and landfills. Some processors recycle these wastes as feed additives for livestock [17]. Some of these wastes include the leaves, spoiled fruits, pulp, unused peels, fibrous materials, and inedible parts of fruits, which comprise the stones, husks, kernels, and, most importantly, the seeds. Swaroopa et al. [18] reported that asparagus, chicory, garlic, onions, honey, wheat, soybeans, peas, beans, bananas, artichokes, tomatoes, and other plant materials are naturally occurring food items that are rich in prebiotics. These naturally occurring foods are also seed-producing.

Technological development over the years has altered the earliest methods of consuming natural foods such as cereals, grains, pulses, fruits, and vegetables. Shahidi [19] and Arulnathan et al. [20] reported that foods such as whole grains and pulses are associated with health benefits. The processing of pulses involves milling, sieving, and debranning, which removes the main functional components [18]. These separated components of grains or pulses have a high oligosaccharide content and can be considered as potential prebiotics. Previous studies revealed that this fraction has a greater prebiotic potential than commercial synthetic prebiotics [21,22]. Prebiotic compounds such as xylo-oligosaccharides (XOS) and arabinoxylo-oligosaccharides (AXOS) are extracted from agro-industrial by-products, including wheat bran, rice bran, rice husk, barley by-products, and finger millet seed coat [21,22,23]. The separated part of the pulses is called the husk, seed coat, or covering [18]. Since there is a reduced amount of prebiotics present in food items that are consumed in the modern diet nowadays, it becomes imperative to produce more prebiotics for consumption. There is a need to screen and find new sources of prebiotics to promote their uptake and scale them up for industrial production. Several researchers in the past have reported the diverse use and isolation of prebiotics from different sources; this study, however, is unique in that, to the best of our knowledge, no studies have reported the potential applications of seed wastes as prebiotics. The objective of this review is to investigate the prebiotic potential of the seed wastes of fruits, cereals, legumes and pulses, and oily seeds. In addition, the existing scientific information for the incorporation of prebiotics as functional foods and the use of seed wastes as potential prebiotics will be reviewed.

## 2. Prebiotics as Natural Supplement

### 2.1. What Are Prebiotics?

The concept of prebiotics was birthed in 1995. The authors of [24] describe prebiotics as a non-digestible food ingredient that advantageously affects the host by selectively stimulating the growth and activity of one or a limited number of bacteria in the colon, thereby improving the host’s health. Using this definition, a few compounds of the carbohydrates group are categorized as prebiotics. These compounds include short- and long-chain fructans (oligosaccharides and inulin), lactulose, and galactooligosaccharides. Gibson et al. [25] reported that dietary prebiotics were described by the International Scientific Association of Probiotics and Prebiotics (ISAPP) at their 6th meeting in 2008 as a selectively fermented ingredient that results in specific changes to the composition and/or activity of the gastrointestinal microbiota, thereby conferring benefits upon the host’s health. Several definitions of prebiotics have been exploited in the literature. Overall, there exists no universal description for prebiotics; however, the relatedness of prebiotics consumption with human health remains the major keyword of both the initial and subsequent definitions [24]. Bindels et al. [26] revealed that “selectivity”, or the efficacy of prebiotics in promoting the growth of some microflora in the gastrointestinal tract, was a part of the first definition that has recently become controversial.

### 2.2. Criteria for Classifying Compounds as Prebiotics

Gibson et al. [27] posited the following criteria as being a necessity in the classification of a compound as a prebiotic. These include being resistant to the acidic pH of the stomach, hydrolyzed by mammalian enzymes, absorbed in the gastrointestinal tract, fermentable by intestinal microbiota, and able to selectively stimulate the growth and activity of intestinal bacteria. It is noteworthy to state that not all prebiotics are carbohydrates as some are of fiber origin. Carbohydrate-derived prebiotics can be distinguished from fiber-based prebiotics via two criteria: fibers are sugars with a polymerization degree (DP) that is higher than or equal to 3 and they cannot be hydrolyzed by the endogenous enzymes in the gastrointestinal tract. It is essential to know that the solubility or fermentation ability of fibers is not outstanding [28,29]. The sources of the pectic oligosaccharides (POS) determine the significant differences in their structures [30].

### 2.3. Types of Prebiotics

Prebiotics are of several types. Carbohydrates (oligosaccharides) make up the largest percentage of the total number of prebiotics [24]. Oligosaccharides have been reported by several studies; however, it is noteworthy that other types of prebiotics exist that are not carbohydrates. The types of prebiotics, with specific examples, are presented in Table 1.

#### 2.3.1. Fructans

Fructans comprise inulins and FOS (also known as oligo-fructose). Structurally, fructans consist of fructose chains arranged in a linear form, with β (2, 1) linkage. The degree of polymerization (DP) is approximately 60 for inulin and less than 10 in FOS [31]. The existence of FOS has been documented in about 36,000 plants by Havenaar et al. [38], even though the concentrations as reported in present plants are relatively low for exerting prebiotic efficacy. Previous studies have established the possibility of selective growth promoting the effects of fructans on LAB. Nevertheless, Scott et al. [32] posited that there have been some studies in recent years revealing the chain length of fructans as a significant condition in determining which bacteria are capable of fermenting them.

#### 2.3.2. Galactooligosaccharides

Galactooligosaccharides have been recognized globally as a prebiotic after several in vitro and in vivo studies on animals and human beings [39]. GOS are generated from the extension of lactose, a disaccharide sugar found in milk and other products. They can be subdivided into GOS with excess galactose attached to the third, fourth, or sixth carbon atoms, and GOS that are generated through the enzymatic trans-glycosylation of lactose.

A mixture of pentasaccharides having a DP of 3–5, with galactose in β (1, 6), β (1, 3), and β (1, 4) linkages, is the end-product of the trans-glycosylation of the lactose reaction [24]. This type of GOS is also known as a trans-galactooligosaccharide (TOS) [27,40]. GOS can promote the growth of Bifidobacteria and Lactobacilli. High populations in infants have been traced to the incorporation of GOS in their diet [24]. Bacteroides and Firmicutes have been reported to be stimulated by GOS, but this is to a lesser extent when compared with probiotics, e.g., Bifidobacteria [31]. An isomer of lactose, known as lactulose, can also be a source of some GOS. Lactulose is an artificial disaccharide with a molecule of galactose and fructose linked together by a bond that is not digestible by lactase. Lactulose resists digestion in the upper gut and it is not absorbed by the small intestine; however, it undergoes fermentation to yield SCFAs, carbon dioxide, and hydrogen, which consequentially results in reducing the fecal pH [41].

Lactulose-derived GOS are also considered to be prebiotics [27]. Other types of GOS are based on sucrose, known as raffinose-family oligosaccharides (RFO). Johnson et al. [34] and Whelan [42] commented that the effect of RFO on gut microbiota has not yet been evaluated.

#### 2.3.3. Starch- and Glucose-Derived Oligosaccharides

There is a particular form of starch with the ability to confer resistance to digestion in the upper gut; this is known as resistant starch (RS). In their study, Fuentes-Zaragoza et al. [33] reported that the production of a high level of butyrate by RS confers health-promoting benefits; hence, it has been recommended for use as a prebiotic. The presence of high RS concentrations has been reported to promote the maximum incorporation of different bacteria of the phylum Firmicutes [43]. *Ruminoccocus bromii*, *Eubacterium rectale*, *Bacteriodes thetaiotaomicron*, and *Bifidiobacterium adolescentis* have been demonstrated via an in vivo study to degrade RS [24]. However, Ze et al. [44] reported that the degradation of RS in mixed bacterial and fecal incubations is not possible in the absence of *R. bromii*. An example of glucose-derived oligosaccharides is polydextrose. It consists of a glucan with branches and glycosidic linkages. There is some evidence that it can stimulate the development of Bifidobacteria [44].

#### 2.3.4. Pectin Oligosaccharides

Pectin, which is a polysaccharide, can also serve as a source of some oligosaccharides [24]. These oligosaccharides are referred to as pectin oligosaccharides (POS). The extension of galacturonic acid and rhamnose, otherwise known as homogalacturonan and rhamnogalacturonan I, respectively, forms the basis of their production [24]. Davani-Davari et al. [24] also reported that the carboxyl groups may be substituted with methyl esterification, causing the acetylation of the structure at carbon 2 or 3. The side chains are connected to ferulic acid or sugars, such as xylose, arabinose, and galactose [35]. The source of the POS determines the significant differences in their structures [30].

#### 2.3.5. Miscellaneous

It is inarguable that carbohydrates meet the stated criteria of prebiotic definitions. There are other substances that do not possess the characteristics of carbohydrates but fit into the prebiotic definition, these compounds are known as non-carbohydrate oligosaccharides. An example of these compounds includes the flavanols derived from cocoa. Flavanols have been the focus of experiments in vivo and in vitro to promote the growth of LAB [45]. Dietary polyphenols have been documented to initiate interaction with gut microbiota to selectively promote or inhibit microbial growth or proliferation [46]. These polyphenols rely on the diverse and specific nature of these gut microbiota to synthesize the secondary bioactive metabolites that are used in human biochemical pathways [47]. As with other polyphenols, cocoa-derived polyphenols have been documented to modulate microbial diversity, either by promoting the growth of beneficial bacteria or by inhibiting pathogenic bacteria, hence providing a prebiotic effect in the gut [25,46,47]. Al-Thubiani and Khan [37] reported that any dietary ingredients, such as non-digestible carbohydrates, lipids, or proteins that reach the colon could potentially be considered prebiotics. We assume that these lipids and proteins could be glycolipids and glycoproteins, respectively.

### 2.4. The Sources and Production of Prebiotics

Prebiotics have been reported to fulfill an essential role in overall human health. Natural food products, such as garlic, onion, sugar beet, asparagus, beans, peas, bananas, barley, chicory soybean, milk, wheat, honey, rye, and, more recently, seed wastes have been revealed to be a good source of prebiotics [48]. Their production has been scaled up due to their low levels in food products, using lactose, sucrose, and starch as raw materials in the production of prebiotics [49,50].

**Figure 1:**
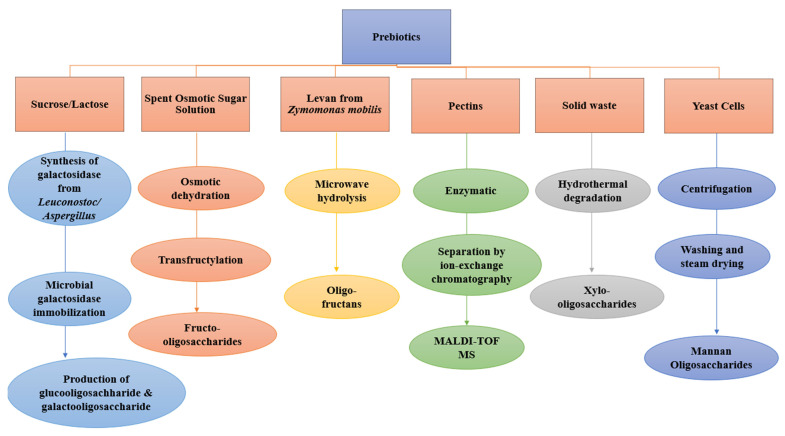
Different methods of producing prebiotics.

Presently, the production of prebiotic oligosaccharides is restricted to their extraction from plant materials, the hydrolysis of polysaccharides by acids or enzymes, or synthesis by trans-glycosylation. The various techniques utilized for producing prebiotics are presented in Figure 1. Patel and Goyal [1] posited that Leuconostoc fermentation, in circumstances where polymer size is restricted, by adding maltose and galactose has also been explored for the production of prebiotics. The chromatographic separation of sugar beet pectin that was degraded by enzymes into several homogalacturonide and rhamnogalacturonide oligosaccharides with a high level of purity was achieved using ion-exchange chromatography. The size and structural features of prebiotics have also been evaluated using MALDI-TOF/TOF mass spectrometry [1].

Holck et al. [51] documented that POS with slight structural differences possess unique biological effects that are significant. β-Galactosidase derived from *Aspergillus oryzae* was immobilized by various methods to produce GOS from lactose at high concentrations. Huerta et al. [52] reported that in optimal conditions, a 30% conversion rate was achieved. In the batch production, 8500 g of GOS per gram of enzyme preparation was produced after ten batches, the yield of which was further expected to increase by replacement with a biocatalyst [52]. *Thermoanaerobacter ethanolicus* JW200 was utilized to produce recombinant α-glucosidase, which was bioengineered and was demonstrated to inhibit *E. coli* growth in the presence of maltose, revealing high trans-glycosylation activity. The products generated from this process were found to be isomaltooligosaccharides, which were classified as a prebiotic [53]. The evaluation of endo-inulinase, which was synthesized commercially from *Aspergillus niger* immobilized on chitin to produce FOS from inulin, has been previously reported in an earlier study by Nguyen et al. [54]. Similarly, *Xanthophyllomyces dendrorhous* 269 has been reported to have the capability of producing fructofuranosidase, an enzyme with high trans-fructosylation activity with prospective uses in the industrial synthesis of neo-FOSs prebiotics, according to Chen et al. [55]. The production of thea-glucosidase, synthesized by *Bacillus licheniformis* TH4-2, was experimentally tested via the transfer reaction of glucosyl to produce a trisaccharide oligosaccharide. The hydrolytic resistance of the product to the intestinal enzymes of rats was responsible for identifying its prebiotic potential [56].

The hydrolysis in a microwave oven of Levan (a high molecular-weight carbohydrate polymer consisting mainly of (2→6)-linked ß-D-fructofuranosyl units) synthesized from *Zymomonas mobilis* and used to generate beneficial oligofructans, which were used to selectively stimulate probiotic bacteria in the gastrointestinal tract, was reported by de Paula et al. [57]. This hydrolysis of levan involved the rupturing of yeast cells through centrifugation, after which the yeast extracts were removed. This was followed by the washing, drying, and pasteurization with a steam drum dryer of the yeast cells to recover prebiotics known as mannan oligosaccharides. These major prebiotics have been classified to be GOS and FOS; hence, most studies reporting the production of prebiotics were mainly centered on their analysis.

#### 2.4.1. Production of Fructo-Oligosaccharides

Sangeetha et al. [58] and Yun [59] reported that various authors have proposed different methods of synthesizing FOS. The chemical production of FOS can be achieved using glycosidase and glycosyl-transferase [60]. This chemical production has the drawbacks of expensive raw materials and the generation of toxic compounds with poor levels of FOS recovery; hence, there is the need for industrial scaling-up [61].

Fructosyltransferase (FTase) [EC 2.4.1.9] is an essential enzyme required for the synthesis of FOS. It is available in many microorganisms that utilize sucrose as their carbon source, including *Fusarium* sp., *Aspergillus* sp., *Aureobasidium* sp., *Penicillium* sp., *Arthrobacter* sp., *Zymomonas mobilis*, *Bacillus macerans*, *Candida kluyveromyces*, and *Saccharomyces cerevisiae* [59,62,63,64,65,66]. Of the above-mentioned microbes, *Aspergillus* *niger* and *Aureobasidium pullulans* have been demonstrated to have the capability of producing FOS on an industrial scale [67]. The biomass or free enzyme can be utilized for FOS production [58,62,63]. The initial sucrose concentration is a factor that determines the maximum amount of FOS to be produced by FTase. The elimination of residues of glucose or fructose is essential during trans-glycosylation because of their inhibitory activities, which consequentially results in a low yield of FOS [58,68]. Previous studies posited that glucose oxidase and β-fructofuranosidase, synthesized from *Apostichopus japonicus* and *Aspergillus niger*, respectively, can be utilized to improve fructo-oligosaccharide yield up to 98% [59,68,69,70].

Sucrose can be converted to FOS by β-fructofuranosidase (FFase) [EC 3.2.1.26]; hence, accumulated glucose from the fermentation process is converted by glucose oxidase to gluconic acid, which can be eliminated by ion-exchange resins or via coagulation using calcium carbonate [70]. Monosaccharides such as fructose and glucose and disaccharides such as sucrose can be removed by *Saccharomyces cerevisiae* and *Zymomonas mobilis* via fermentation, by transforming them into ethanol and CO_2_. However, the inability of *Saccharomyces cerevisiae* to ferment oligosaccharides containing four or more units of simple sugars (monosaccharides) is well documented. The production of sorbitol and fructo-oligosaccharides in small quantities by *Zymomonas mobilis* during the fermentation of sucrose has been reported in previous studies [71,72,73,74].

#### 2.4.2. Galactooligosaccharides

Chemical synthesis by nucleophilic and electrophilic displacement was adopted initially for producing GOS but was found to be uneconomical on a large scale [75,76]. Galactosyl-transferase and galactosidase are the most important enzymes required for synthesizing galactooligosaccharides. Galactosyl-transferase is a stereoselective bio-catalyst capable of synthesizing galactooligosaccharides in large quantities [76]. Although the biological production of GOS using galactosyl-transferase has been reported not to be cost-effective because of the nucleotide sugars required as a donor, an alternative method for decreasing the cost was suggested to be globotriose production [75,77] or the utilization of human milk oligosaccharides [78,79].

The use of galactosidase, a less stereospecific enzyme, to produce GOS is more economical than galactosyl-transferases even though a lower concentration of GOS is produced. The optimization of the quantities of GOS produced can be achieved via increasing the concentrations of donors and acceptors, reducing the water activity and adjusting the equilibrium of the reaction to the end-product through product removal and distortion of the conditions of production [75,80]. *Aspergillus oryzae*, *Sterigmatomyces elviae*, Bifidobacteria, and Lactobacilli are various sources of β-galactosidases. These sources have been posited to have a significant impact on the relevant variables (concentration, DP, optimizing conditions, and glycosidic linkages) [81,82,83,84]. Sources from molds and bacteria, as well as yeast, for instance, require acidic and neutral pH, respectively. Additionally, thermophilic sources require a high degree of temperature [81,85,86,87].

Fukuda et al. [88] reported that the whole cell of the microbe is utilized for GOS production when the isolation procedure of β-galactosidase is too expensive. Whole-cell utilization has been shown to be insignificant in terms of producing GOS because of the metal ions used as co-factors by β-galactosidase, even though whole-cell use has been revealed to be cost-effective owing to the naturally occurring co-factors in the cell [89,90]. Glucose and galactose are by-products of GOS without any records of having prebiotic potential; hence, they have significant effects on the yield by decreasing the amount of GOS. These by-products can be eliminated by other metabolic activities when a whole cell is utilized. If lactose medium is exploited for the culturing process during the production of GOS, *Sirobasidium magnum*, *S. elviae*, and *Rasopone minuta*, for example, can generate their carbon source using glucose [91,92,93,94]. Ethanol, lactic acid, and acetic acid are other by-products generated when whole cells are used for synthesizing GOS, which has a negative influence on the production of GOS; hence, their removal using other methods is necessary.

When non-thermophilic cultures are used when producing GOS, temperature becomes a limiting factor; even though temperature favors the production of GOS, it is highly detrimental to microbial cells. Previous studies have posited the use of non-viable and resting cells that can produce a greater quantity of GOS and do not have any known limitation, as in the whole cell [72,81,95]. High product recovery, ease of purification, enhanced stability, and the activity of the bio-catalyst through molecular approaches are the advantages of recombinant β-galactosidases over indigenous β-galactosidases [96]. Recombinant β-galactosidases are usually synthesized from *Escherichia coli* and *Bacillus subtilis*, even though the former has some setbacks, with known toxic effects including the production of endotoxin and the complications arising from acetate production and disulfide bond formation [97,98]. The bio-engineered *Bacillus subtilis*, on the other hand, does not produce any type of toxin but is known for plasmid instability and protease production [57,98]. *Saccharomyces cerevisiae* and *Pichia pastoris* are yeasts exploited for their potential in generating the recombinant types of β-galactosidases. High yield, the generation of disulfide bonds, and enhanced protein folding are all merits of yeasts compared to bacteria [98,99,100].

### 2.5. Assessment of Prebiotic Efficacy

The study on the different techniques used in the assessment and evaluation of prebiotic efficacy can be demonstrated in three phases, in terms of digestion, fermentation, and analysis [101]. The assessment of the non-digestibility of a prebiotic should be evaluated. Resistant to gastric acidity, mammalian enzyme hydrolysis, and absorption by the small intestine are the characteristics of an ideal prebiotic [102]. To conduct this type of evaluation, an undefined microbial media containing yeast extract, sodium chloride, calcium chloride hexahydrate, magnesium sulfate heptahydrate, water, Tween 80, 1-cysteine hydrochloride, vitamin K, haemin, resazurin, and bile salts was developed [103]. Both the digestion and fermentation phases can be studied using in vitro and in vivo techniques. Table 2 summarizes the types of techniques and their conditions of assessment for the in vitro, animal, and human models for assessing the efficacy of prebiotics.

Evaluation of the digestion of prebiotics is aimed at imitating the human digestive tract at the different phases of digestion [106]. The oral phase is established for 5 min at pH 7 in the presence of NaCl and alpha-amylase [104]. Conversely, the gastric phase is conditioned at pH 2.5 for 2 h in the presence of the digestive enzymes (pepsin and gastric lipase) in the stomach. Once the gastric phase has ended, the digestion of prebiotics can then be examined in the final phase (small intestine) of the digestion process [105]. Enzymes such as chymotrypsin, trypsin, colipase, and pancreatic lipase are introduced for 2 h at pH 6 in the small intestine phase [106]. To evaluate the digestibility of prebiotics using in vivo techniques, the use of animal models, specifically rats and piglets, have been documented in several studies to be appropriate for these purposes [121]. When animal models are used, digestibility is tested by measuring the digestion of the prebiotic in the fecal sample; intubation of the prebiotic in the GIT of the experimental animals can thereby be estimated [106]. Similarly, when human clinical studies are conducted, an estimation of the undigested prebiotic in the distal ileum is carried out [106].

The in vitro fermentation of prebiotic compounds by colonic microflora is achieved via three fermentation systems, including a batch culture fermentation system, continuous fermentation system and the artificial gut system [122]. A pH-based batch culture is used for the batch culture fermentation system. In this system, the pH of the fermenter is sustained at the appropriate level through the use of the gas production technique during the fermentation process [108]. However, continuous culture fermentation could either be single- or multi-staged, depending on the type of fermentation [106]. Two different models, including TIM (The Netherlands Organization for Applied Scientific Research intestinal model) and SHIME^®^ (the simulator of the human intestinal microbial ecosystem), are employed for the in vitro study of prebiotic fermentation in the artificial gut system [113]. Just as in the in vivo assessment of prebiotic digestion, animal and human models are used for the in vivo evaluation of prebiotic fermentation. Following the administration of prebiotics to the animal models, fecal samples are collected, and the measurement of the fermentation level of the candidate prebiotics is conducted. However, in a human clinical study, prebiotic evaluation can be achieved indirectly via fecal collection or directly by collecting the breathed air [113].

Assessment of the prebiotic efficacy is completed after the final stage, which involves analyzing the SCFAs (the fermentation products of carbohydrates by gut microflora) and evaluating the increase in the gut microbiota using different molecular techniques [123]. Generally, the SCFAs directly stimulate the growth of the gut microbiota and, consequently, result in an increase in the microbial population in the gut. This relationship has been reported to be detectable by gas chromatography (GC) and high-performance liquid chromatography (HPLC). To enhance the efficiency of detection by GC, it had been reported that flame-ionization detection or mass spectrometric detection can be combined with GC. Reversed-phase and ion exclusion columns are commonly used with HPLC for the detection of SCFAs [106]. Acetylated XOS and XOS efficacies have been studied, using HILIC-ELSD. In one study, a zwitterionic HILIC column was used with an ELSD detector [106]. Xiao et al. [116] reported the determination of structural information of X7, X8, and acetylated XOS using LC–ESI–MS.

Diversity exists in the types of bacteria found in the gut. These bacteria are specific to the fermentation of prebiotics, and this can be detected using modern strategies. Previously, culture-based techniques have been employed to study the gut microbiota in human fecal samples. This technique metamorphosed into a DNA-based culture-independent procedure where a specific quantity of nucleic acids (DNA and RNA) can be detected. The quantity of the DNA is measured using real-time PCR (RT-PCR) or quantitative PCR (qPCR) [117]. Following the measurement of the DNA, the identification of the bacteria is completed via sequencing of the genes using either 16s ribosomal RNA or the pyrosequencing technique [106]. Bacterial populations in the gut are differentiated in 16s rRNA genes by using the phylogenetic signal from different levels of variability [124]. Pyrosequencing works on the principle of light emission from the synthesized DNA. This light is captured and analyzed using computational programs such as the Primer Express software, qbase+ [120].

**Figure 2:**
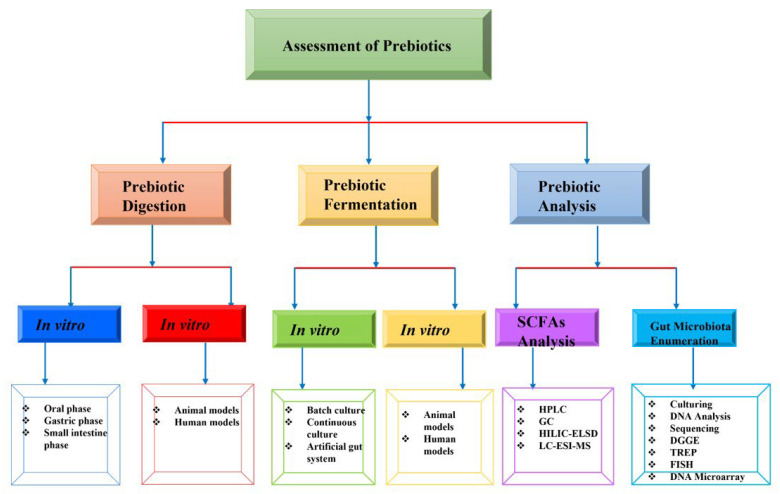
Assessment of the efficacy of prebiotics.

Apart from the aforementioned methods, other techniques have been reported to be applicable in studying the DNA of bacterial populations. Denaturing gradient gel electrophoresis (DGGE) can be used for the identification and characterization of specific DNA patterns as biomarkers [106]. Terminal restriction fragment length polymorphism (T-RFLP) can also be employed to carry out DNA fingerprinting for comparing the gut microbiota’s diversity and variability [119]. DNA microarray technology, equipped with a laser-induced fluorescence scanner that can detect probe-target duplex, has been documented for phylogenetic identification, gene expression, specific DNA sequences, and the determination of mutations in the genes of gut microbiota [106]. Additionally, the use of fluorescent in situ hybridization (FISH) for gut microflora identification has been well documented by earlier studies. In this technique, the bacterial cells are fixed and hybridized on a glass slide and then observed using a fluorescence microscope. A summary of the techniques for evaluating prebiotic efficacy is presented in Figure 2.

### 2.6. Health Benefits and Mechanisms of Prebiotics

**Figure 3:**
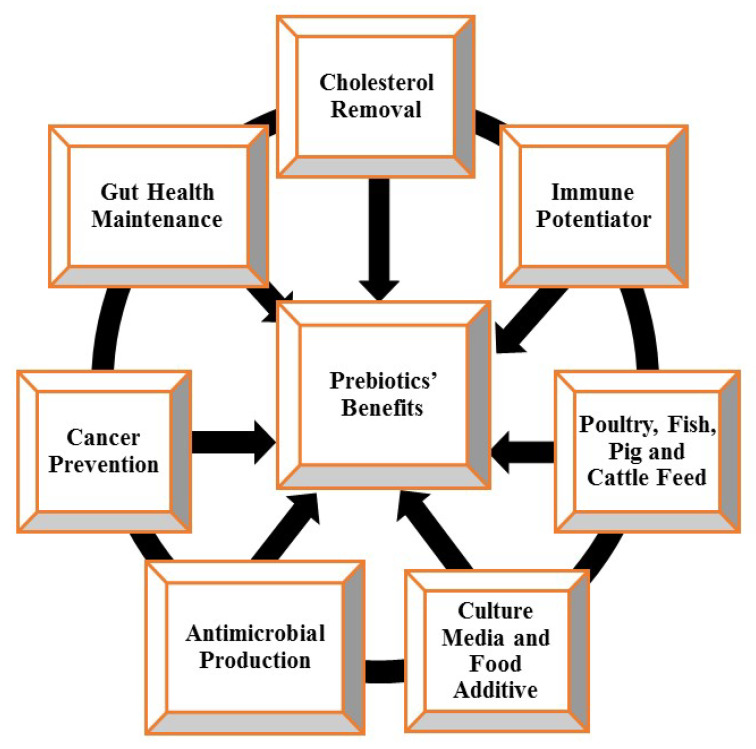
Potential applications of prebiotics.

Prebiotics have been implicated in several beneficial effects on human health by previous studies. The current use of prebiotics is shown in Figure 3.

#### 2.6.1. As Starter Culture Media and Food Additives

The quality of skim milk fermented by the pure or mixed culture of *Lactobacillus acidophilus*, *Lactobacillus rhamnosus*, *Lactobacillus bulgaricus*, *Bifidobacterium lactis*, and *Streptococcus thermophilus* can be enhanced by supplementing with inulin. Oliveira et al. [125] have reported a lower generation time for *Streptococcus thermophilus* and *Lactobacillus acidophilus* by supplementing their growth media with inulin. The lowering of the generation time results in the blooming of beneficial organisms. The influence of FOS and inulin was conducted by Rodrigues et al. [126] on the profile of free fatty acids obtained from cheese. The study placed specific emphasis on conjugated linoleic acid. This study suggested the addition of prebiotics to increase the conjugated linoleic acid content during the ripening stage to make quality cheese with a reduced atherogenic index. The prebiotic influence of cow’s milk-based infant formula enriched with polydextrose on a day-old piglet for 18 days was examined by Herfel et al. [43]. An increased ileum-based LAB population, which consequentially resulted in an increased amount of lactic and propionic acids with a reduced pH, was observed in the experiment. In a single-arm study of nine infants suffering from phenylketonuria (PKU), a genetic disorder characterized by the inability to metabolize phenylalanine in the diet, infant formula that was free of the amino acid as an alternative was evaluated by MacDonald et al. [127] to establish the impact of including an oligosaccharide-based prebiotic compound into a protein substitute feed. PKU Anamix Infant, a diet designed for infants with PKU, has a prebiotic oligosaccharide content of 0.8 g/100 mL with a GOS/FOS blend similar to that in breast milk. It maintained phenylalanine in the control, enhanced the levels of Bifidobacteria, and lowered the pH [1].

#### 2.6.2. Gastro-Intestinal Tract (GIT) Improvement

The α-galactosidase activity of various probiotics, such as *Lactobacillus acidophilus* FTDC 8033, *Lactobacillus acidophilus* ATCC 4356, *Lactobacillus casei* ATCC 393, *Bifidobacterium* FTDC 8943, and *Bifidobacterium longum* FTDC 8643, has been reported to be improved through the supplementation of soy milk with FOS and maltodextrin, thereby improving the hydrolysis and utilization of glucose and fructose, to promote their growth, and preventing colitis as well as constipation [7]. Patel and Goyal [1] reported inulin, FOS, mannooligosaccharides, and arabinogalactans as therapeutic nutritional preparations that can be utilized for optimal functioning of the GIT, as well as for promoting the growth of the microflora and simultaneously causing the limitation of pathogenic microbes. The consumption of prebiotics can modulate immune parameters in gut-associated lymphoid tissue (GALT), secondary lymphoid tissues, and the peripheral circulation [128]. Srinivasjois et al. [129] reported that an increased count of Bifidobacteria and lactobacilli colonies in the stools was observed in their study of preterm neonates, without negative effects on weight gain, when the infants were fed with infant formula supplemented with prebiotics. The consumption of infant formula containing prebiotics increases the fecal bolus and the frequency of depositions, as well as reducing constipation, which is a resultant effect of the lack of fiber in the diet of neonates [3].

#### 2.6.3. Anticancer Agents and Immune Potentiators

The consumption of a diet fortified with arabinoxylan-oligosaccharides (AXOS) was reported to cause a reduction in preneoplastic lesions in the gastrointestinal tract of rats treated with a carcinogen [1]. *Ruminococcus albus* has been reported to effectively convert lactose to epilactose by producing cellulobiose 2-epimerase. Improved Lactobacilli and Bifidobacteria colonies, the increase and decrease of cecal content and pH, respectively, and Clostridia or *Bacterioides* suppression in Wistar-ST rats have been traced to epilactose-supplemented diets [1]. The conversion of primary bile acids to secondary bile acids, which are colon cancer promoters can be impeded by prebiotics [130]. Propionate has been revealed to confer an anti-inflammatory benefit to cells in the cancer-infected colon [131]. The suppressed expression of transcription factor Nuclear factor-kappa B (NF-KB) in HT-29 cell lines by butyrate and increased peripheral blood antibody production and natural killer (NK) in cancer patients have been evaluated [40]. Neoplasia, diabetes mellitus, and coronary diseases are examples of degenerative disease incidences that have been reported to be reduced by prebiotics. Prebiotics also seem to exert a positive modulation of the immune system [132]. Elsewhere, the antibody reactions of neonates to influenza and tetanus vaccinations in their first year of life was evaluated by Stam et al. [133]. The experiment was conducted by observing the effects of prebiotic compounds that were supplemented in the infant formula. Short-chain galactooligosaccharides, long-chain fructo-oligosaccharides, and pectin-based acidic oligosaccharides have been posited to be similar in composition to the oligosaccharides present in mammalian milk. This mixture promotes T-Helper 1 and regulatory T-cell-dependent immunological responses and induces the downregulation of IgE-mediated allergic responses. Overall, interference of the prebiotic with the desired antibodies in the control group (healthy full-term infants) was not observed [133].

#### 2.6.4. Removal of Cholesterol, Cardiovascular Disease Reduction, and Obesity Prevention

The risk of coronary diseases and vascular disorders has been reported to be reduced through the consumption of resistant carbohydrate-rich whole grains [134]. An increase in plasma ferulic acid has been associated with the concurrent increase in free ferulic acid from enzyme-treated prebiotic durum wheat and has been reported to be the cause of the health benefits of dietary fiber in patients with cardiovascular diseases [135]. Hess et al. [136] reported that satiety can be induced and consequently prevent obesity by supplementing dietary fiber, such as short-chain fructo-oligosaccharides, which can undergo fermentation. The effects of prebiotics in diet supplementation on satiety were evaluated by Cani et al. [137] using a randomized controlled trial experiment for 14 days with ten healthy humans as subjects. The research concluded that prebiotic treatment and the consequent hunger reduction were associated with an increase in the postprandial concentration of the relevant peptide. The monitored consumption of food and the regulation of glucose as a way to lower the risk of obesity has been associated with prebiotic intake. The effect of a soy food diet enriched with prebiotics in adults suffering from hyperlipidemia has been investigated. The consumption of prebiotics fortified with soy revealed a decrease and increase in the concentration of low-density and high-density lipoprotein cholesterol, respectively. The improved effectiveness of the soy diet serum profile may be potentiated by the co-ingestion of a prebiotic [1].

#### 2.6.5. Vaginal Ecosystem Restoration

Anaerobic pathogens have been reported to take over the normal flora of the vagina in the post-menopausal period in women. To combat this, the co-consumption of prebiotics with probiotic organisms has been proven to restore the vaginal microbiota [1]. A vaginal bio-adhesive delivery system was created using pectinate-hyaluronic acid microparticles for probiotic and prebiotic encapsulation by Pliszczak et al. [138].

#### 2.6.6. Production of Antimicrobials

*Pediococcus acidilactici* LAB 5, isolated from vacuum-packed fermented meat products, possesses the ability to produce a prebiotic known as sorbitol, which positively influences bacteriocin production [9]. Inulin, raffinose, and lactulose prebiotics were studied by Vamanu and Vamanu [8] for their effects on the production of bacteriocin from the *Lactobacillus paracasei* CMGB16 strain. The positive result indicated by *E. coli* inhibition was established by an agar-well diffusion technique.

#### 2.6.7. Production of Environmentally Friendly Agricultural Feeds

An increase in the consumer perception of antibiotics usage and residues has caused a paradigm shift to alternative sources [1]. *Salmonella typhimurium* and *Salmonella enteritidis* are major causes of foodborne illnesses and infections in humans and poultry, respectively. The increased phagocytic and destruction activities of macrophages have been observed in vivo in the dose-dependent treatment of the MQ-NCSU chicken macrophage cell line with the prebiotic β-1,4-mannobiose (MNB) [1]. Similarly, a significant increase in the genes responsible for host immunity and antimicrobial activity expression has been revealed by the gene expression analysis of MNB-treated macrophages [139]. Xu et al. [4] revealed the enhanced growth and digestive enzyme activities of the allogynogenetic crucian carp (*Carassius auratus gibelio*) by prebiotic xylooligosaccharides. Salmonid aquaculture using probiotics and prebiotic applications has achieved tremendous breakthroughs, such as enhanced health status, increased growth performance, disease resistance, better body composition, gut microflora balance and morphology, and decreased malformations. The effects of various inulin prebiotic levels on the hematologic and biochemical parameters and the blood enzymes of the juvenile great sturgeon were evaluated by Ahmdifar et al. [11]; it was concluded from the results that there is a significant increase in enzymes and white blood cell count with an increasing level of inulin supplementation. The presence of lactobacilli was observed in Holstein heifer calves when fed with a prebiotic supplement [140]. The development of hemorrhages was observed in dairy cattle fed with mycotoxin-producing fungi-contaminated feed [1]. The condition was treated with a Celmanax prebiotic formulated using the cell walls of non-living yeast that are capable of producing anti-adhesive activity for the Shiga toxin producing *E. coli* O157:H7. Improvement in milk production and feed conversion and the reduction of mycotoxin in cattle were observed after the treatment [141].

#### 2.6.8. Prebiotic Supplementation Provides Nutritional Value

Cyanobacteria such as *Spirulina* sp. have been found to offer high nutritional value, being a rich source of amino acids, proteins, calcium, vitamins A, B2, B12, E, H, K, essential minerals, iron, x-6 fatty acids, and trace elements [1]. An aqueous suspension of *Spirulina platensis* dry biomass was reported by previous studies to have a stimulatory effect on four lactic acid bacteria isolated from milk. The inclusion of dry *Spirulina platensis* in milk (6 mg/mL) stimulated about 27% growth of *Lactococcus lactis* [142]. The addition of *Spirulina* sp. biomass to fermented milk to induce lactic acid bacteria in the product and gut is being considered in the dairy industry, rather than supplementation with minerals, vitamins, and antioxidants. Angioloni and Collar [87] reported that high-viscoelasticity dietary fibers imbue bread with better organoleptic properties, a reduction in digestible starch concentration, and an increased resistant starch content, thereby reducing the glycemic index in vitro. The in situ generation of arabinoxylan-oligosaccharide prebiotics during breadmaking was investigated by Damen et al. [143]; it was reported that the bread’s quality was improved by the cleaving action of xylanase on the arabinoxylan fraction of the cereal by the *Hypocrea jecorina* fungus. This resulted in arabinoxylan-oligosaccharides production, with 2.1% content in the fortified dough. The growth of *Lactobacillus johnsonii* B-2178 was used to examine the prebiotic impact of oligosaccharides containing fermented cashew apple juice; the study concluded that growth in the fermented cashew apple juice was threefold that of the observed growth in the non-fermented juice [144]. Milk whey culture has been suggested by Uchida et al. [145] to be an important prebiotic for treating inflammatory bowel disease. Ice creams supplemented with *Lactobacillus casei* and 2.5% inulin showed good nutritional and organoleptic qualities [146].

Table 3 provides a summary of the uses of different prebiotics, with their respective functionality across different industries.

## 3. Seed Waste as a Source of Prebiotics

Waste was described by the Waste Framework Directive 2008/98/EC laws as any substance or object that the holder discards or intends to or is required to discard. Seed wastes are organic substance containing spent grain, rice husk, maize husk, wheat husk, walnut shell, coconut shell, and groundnut shell. Culturally, seed wastes are discarded in landfills to rot, consequently causing soil, water, and air pollution, as well as acting as vehicles for pathogens [147]. Generally, seed wastes are biodegradable and contain essential compounds, such as polysaccharides, dietary fibers, and vitamins. They can be employed as a cheap and alternative source of important food products such as prebiotics [147]. Currently, there is increasing interest in the investigation, identification, and development of new prebiotic sources to support the claim of using functional foods as an alternative approach for health promotion and a reduction in the risk of diseases [147].

One common waste of soybean seed processing is whey. Patel and Goyal [1] reported that soybean whey generated from tofu, which is usually discarded, contains non-digestible oligosaccharides (NDOs). An increase in the calcium and magnesium absorption in the caecum has been associated with the acidic fermentation of NDOs; hence, the whey could be utilized as an essential raw material in functional foods [148]. Similarly, Nimpiboon et al. [56] reported the growth-stimulating effect of soy sauce lees oligosaccharides (SSLO) as a prebiotic on *L. bulgaricus* and *S. thermophilus*. The exposure of water-extractable polysaccharides isolated from wheat bran and Bengal husk to driselase hydrolysis will result in the production of oligosaccharides [149]. The trans-fructosylation of spent osmotic sugar solution, generated from the osmotic dehydration of carrot cubes, has been reported by Aachary and Prapulla [69] to be a source of FOS. Barley husk, spent grains, and grain fragments are solid wastes generated from the malting of barley, which can be processed into xylooligosaccharides containing liquor through hydrothermal techniques [1]. Wang et al. [150] reported that mung beans may improve *Lactobacillus paracasei* growth. The simultaneous saccharification and solid-state fermentation of the refined product of apple pomace, which is rich in pectic oligosaccharides, was studied by Gullon et al. [151] for its prebiotic potential. Smith et al. [152] concluded that a novel legume-based food ingredient called lupin kernel fiber is prebiotic. It has the capacity to modulate the microbiota in the human colon, which was evident from a significant increase in the count of *Bifidobacteria* sp., with a simultaneous decrease in the counts of *C. ramosum*, *C. spiroforme*, and *C. cocleatum*. Table 4 highlights the different seed wastes and their potential functions as prebiotics.

### 3.1. Fruit Seeds

#### 3.1.1. Date Seeds

Generally, date seeds are regarded as waste from industries that process dates into powders, pastes, confectionery, syrup, pitted dates, chocolates, and coated dates [37]. Food and Agriculture Organization [174] posited that more than 800 million tonnes of dates were produced globally in 2018, with over 8 million tonnes discarded as waste; hence, the date seed represents a significant quantity of the global generated waste. On average, depending on the grade, variety, and maturity, date seed accounts for 10 to 15% of total date fruit mass [175,176]. Around 10–15% of the date fruit is constituted by the seed or pit. The seed consists of a small embryo and a hard endosperm, and is rectangular and grooved ventrally [177]. Chemically, a date seed contains 60 to 80% fiber and 4 to 14% of oil by weight. In addition, alkaloids, flavonoids, anthraquinone, saponin, terpenoids, and tannins, as well as major plant elements such as potassium and calcium, are present [177,178,179].

Date seeds are rich in phenolic components, such as phenolic acids and flavonoids. Nehdi et al. [180] reported the total phenolics in date pits to range from 3102–4430 mg of gallic acid, while their antioxidant equivalents/100 g fresh weight ranges from 580 to 929 mol Trolox equivalents per gram. They serve as a good source of protein, ranging from 5–6%, including albumin, globulin, prolamin, and glutelin as soluble proteins. Moreover, high amounts of carbohydrates (71–90%) were also reported in some varieties, mainly in the form of insoluble fibers. From these studies, it is evident that date seeds are not a potential source of proteins that are digestible; however, the carbohydrate and lipid profiling of date seeds confirms that they could be further exploited for the development of functional foods [179].

Date seeds have a large quantity of fiber—both soluble and insoluble—that confer their various health benefits. The dietary fiber content in date seeds was as high as 71.5% with the presence of resistance starch, insoluble, and soluble dietary fibers. Date seed carbohydrates are composed mainly of insoluble fiber, at around 50% cellulose and 20% hemicellulose. Date seed endosperms store carbohydrates in the form of β-D mannans with 1,4 linkages. The degradation of mannans is facilitated by the enzymes endo-β-mannanase and β- mannosidase, owing to the release of insoluble mannans into the matrix [181]. Not only dietary fiber but also the higher amounts of phytochemicals found in date seeds make it a good potential functional food ingredient. Carotenoids are major phytochemicals in date seeds, giving them their characteristic brown color. Date seeds have also been reported as potential sources of phenolic acids, flavonoids, phytosterols, etc. An abundance of oleic acid in date seeds also directly contributes to their antioxidant potential. The fatty acid profiling of date seeds indicated the presence of lauric, linolenic, palmitic, and myristic acids [182].

Traditionally, date seeds were utilized as animal or poultry feed, as well as soil fertilizer [183]. However, the presence of the enormous quantity of dietary fiber contained in date seeds qualifies them to be considered prebiotics. Al-Farsi et al. [178] reported that the dietary fiber concentrates obtained from date kernels could potentially be an inexpensive source of natural dietary fiber and probably a functional food ingredient. Various uses of date seeds in both the food and nonfood industries have been documented. Nancib et al. [155] conducted a study on the utilization of date seed components and flesh to cultivate the probiotic *Saccharomyces cerevisiae*. It was reported that date components are a good source of nitrogen and carbon for fermentation, and a high yield of bakers’ yeast was recovered. An increase in crude fiber and minerals in cereal-based snacks and other baked products can be obtained by processing date seeds into powder, which can then be used as a cost-effective ingredient [153,154].

#### 3.1.2. Grape Seeds

Grape seed extract is an excellent antioxidant, making them an interesting candidate for functional food development. Their antioxidant potential is explained by the complex chemistry of grape seeds, especially the high levels of phenolic compounds (5–8%, contributed by flavonoids, tannins, stilbenes, and phenolic acids), vitamin E, and phytosterols [184]. The seeds generally contain carbohydrates, lipids, and proteins. However, the components of interest in grape seeds are polyphenols. Flavonoids constitute the major polyphenols in grape seeds. They are found in the form of gallic acid, catechin, epicatechin, gallocatechin, epigallocatechin, epicatechin 3-O-gallate, B1-3-O-gallate, 3-flavonols, procyanidin dimers, trimers, and the more highly polymerized procyanidins [118,185]. Chen et al. [186] reviewed research progress into the bioactivity of grape seeds, highlighting their physiologically active ingredients contributing to their pharmacological and functional properties. Grape seed oil, as with other seed oils, is a component of interest for various researchers. Studies by Karaman et al. [187] reported the protein content of grape seed oil as 9.3% and of crude fiber as 45%. Grape seed flour is also a good source of dietary fiber (40–45%); composition varies, depending on the seed variety and conditions [188]. This strongly seconds the use of grape seed oil as a functional food ingredient by bringing the beneficial effects of dietary fiber. Grape seed pulp powder, when incorporated into ice cream along with pomegranate and sesame seeds, displayed excellent antioxidant properties and prebiotic potential, owing to the presence of dietary fibers. They promoted the growth of probiotic microorganisms and positively affected their survival [189].

Proanthocyanidins with different polymerization degree values can be utilized as nutraceuticals in many products. One of the richest sources of this compound is grape seeds [190]. Proanthocyanidins isolated from grape seeds have been revealed to possess potential as antithrombotic, antitumor, anti-mutagenic, anti-radiation-damage, and antifatigue substances [156,157].

#### 3.1.3. Mango Seeds

The king of fruits, mango is the most celebrated tropical fruit in the world, owing to its appearance, taste, nutrition, and aroma. The data released by Food and Agriculture Organization (FAO) stats in 2015 reported the annual world production of mango to be as high as 40 million tons. Mango processing units across the world have reported that around 35–60% of an individual mango is discarded as waste. The primary source of waste from mango is the seed, which accounts for more than one million tons of annual waste [191,192]. The seed is regarded as a biowaste due to its physicochemical and nutritional profile. The presence of bioactive compounds, including phenolic compounds, carotenoids, vitamin C, vitamin E, vitamin A, and dietary fiber were reported by the authors of [160,193]. Vitamins A, C, and E are considered to be antioxidant vitamins, suggesting that due to their presence in mango seeds, the seeds could act in reducing oxidative processes/stress, directly conferring health benefits. This was confirmed and reported by the authors of [194], who listed the ethanolic extract from mango seed as one of the top four extracts with increased antioxidant potential. Maisuthisakul and Gordon [158] reported that mango seed kernel extracts were rich in polyphenols, in the form of gallotannins and condensed tannin. This also contributes to the prebiotic potential of mango seeds as polyphenols play a significant role in gut microbiota modulation [159]. They influence intestinal microbial growth, directly interfering with cell function.

#### 3.1.4. Tamarind Seeds

Tamarind seed polysaccharides (TSPs) are storage units in the seeds and are extracted by acid and alkaline hydrolysis. TSPs are composed of glucose, galactose, and xylose sugar monomers, constituting 65% of the total seed components. Studies have reported that TSPs offer antidiabetic activity by lowering blood sugar levels [162]. They also exhibit gel-forming ability when used along with sugar or alcohol, making them a potential ingredient that can form pectin resembling the gels in food matrices [195]. Tamarind seed extracts also display antimicrobial and antioxidant potential. It was confirmed that tamarind seeds act as prebiotics for the growth of lactic acid bacteria when incubated at 70 °C for 180 min [161]. The presence of the xylose sugar monomers in tamarind seed kernel makes them an effective replacer for food-grade starch [196]. A functional yogurt conferring prebiotic potential was developed using tamarind seed kernel powder, proving the same findings.

### 3.2. Cereals

Globally, a significant portion of the human diet is made up of cereals and grains, which include wheat, rice, barley, maize, sorghum, millet, oats, and rye [197]. Anal [198] posited that many by-products (e.g., bran and germ) are generated from cereal processing. One of the most essential sources of dietary fiber in humans is from cereals [163].

Dietary fibers are carbohydrate polymers containing more than 10 monometric units that resist digestion by the endogenous enzymes of the small intestine [199]. Cereal-based dietary fibers have been documented by several studies for their health benefits, such as satiety regulation and the dilution of the energy density of food. Several studies have also documented the prebiotic activity of cereal-based dietary fiber components such as β-glucans and fructans. Similarly, some studies have examined wheat-based arabinoxylan prebiotic activity [200,201,202,203]. An increase in stool weight and bacteria-holding capacity has been associated with the inclusion of insoluble dietary fibers, especially cereal-based, in the human diet [163]. Similarly, a reduction in the glycemic index of food, insulin sensitivity, cholesterol absorption, and colorectal cancer has been reported for soluble dietary fibers [163].

One of the most abundant and yet underutilized by-products is rice bran, generally obtained from the outer layer of the rice grain [164,204,205]. It is an essential source of dietary fiber, including soluble and insoluble fibers, such as arabinoxylans, hemicellulose, lignin, and β-glucans. Since these compounds resist digestion and absorption in the small intestine, they are fermented by the colonic microbiota and consequently increase fecal bulk through the proliferation of bacteria, as well as microbial metabolites, such as short-chain fatty acids and gas formation [206,207,208]. Soluble, viscous, and fermentable dietary fibers have been reported to enhance human health, gaining a reduction in the glycemic index, insulin sensitivity, a reduction in cholesterol absorption, and the prevention of diseases [209,210].

#### 3.2.1. Brewer’s Spent Grains (BSG)

A fiber-rich waste product of beer-producing factories is known as brewer’s spent grains (BSG), which can be produced at low cost and high volume [211,212,213]. It contains an approximately 42% xylose-based polymeric or oligomeric material—hence, its utilization as an ingredient for producing arabinoxylan-oligosaccharides (AXOSs) [214]. BSG is a heterogeneous substance, owing to the variation in composition due to barley variety, harvesting time, malting, and mashing, as well as the quality and type of the adjuncts included during the brewing process [166].

BSG, owing to their composition and bioavailability, are considered a prime source of lignocellulosic biomass [215]. Sugars contribute to the major fraction of the BSG, nearly 50% on a dry-weight basis. BSG is reported to be a rich source of carbohydrates (30–70%), fermentable sugars, lignins, and protein (15.3–24.7%) [216,217]. Among the cellulose and hemicellulose fractions of sugars in BSG, arabinose and glucose are in abundance [218]. The percentage composition of polysaccharides in BSG are reported as hemicellulose (192–400 g/kg), cellulose (168–260 g/kg), lignin (119–278 g/kg), protein (153–247 g/kg), and starch (0.28–8%) [215,219]. In total, 30% of the total protein content of BSG is from the essential amino acids, especially lysine, contributing to 14.31%, followed by leucine 6.12%, phenylalanine, isoleucine, and threonine, at 4.64, 3.31, and 0.71%, respectively. Among the non-essential amino acids, histidine, glutamic acid, and aspartic acid represent the major fractions, at 26.27, 16.59, and 4.81%, respectively [220,221].

Numerous health benefits, including accelerated transit time, increase in fecal weight, increase in fat excretion, reduced gallstones, and plasma cholesterol, as well as postprandial serum glucose level, has been attributed to the consumption of BSG [222,223]. The potential of BSG as a raw material to produce AXOS that are suitable as prebiotics for elderly people has been investigated by Gomez et al. [165]. The presence of the substituted degree of polymerization value of about 2 to 10 and an unsubstituted value of 2 to 16 oligosaccharides, made up of xylose and arabinose, was observed in the purified mixtures. The study concluded that AXOS are better substrates compared to FOS, in terms of the variation in the bacterial count and the production of SCFA.

#### 3.2.2. Coffee Spent Grounds (SCGs)

Coffee, the most popular non-alcoholic beverage, as seen in its excessive consumption, leaves behind derivates in the form of coffee spent grounds (SCGs). Recent studies [224] have reported that an average of 6 million tons of SCGs are generated as coffee processing waste per year. Green coffee beans are rich in carbohydrates and polysaccharides, in the range of 50–54%. This percentage comprises mannans and cellulose [225]. The SCGs, when subjected to delignification and defatting, yield polysaccharides, out of which the major polysaccharides reported in SCGs are of lignocellulose [226]. Studies have also described the presence of monooligosaccharides (MOS) (a nondigestible oligosaccharide comprising mannobiose, mannotriose, and mannotetraose) in SCGs, and directly contributes to the prebiotic potential of SCGs. MOSs are produced by the two-staged enzymatic hydrolysis of SCGs [168]. Pérez-Burillo et al. [169] stated that MOS from SCGs exhibited prebiotic potential, as it stimulated the growth of beneficial gut microbes, such as Barnesiella, Odoribacter, Coprococcus, Butyricicoccus, Intestinimonas, Pseudoflavonifractor, and Veillonella.

#### 3.2.3. Buckwheat

Buckwheat seeds or grains contain a variety of nutrients, including proteins, polysaccharides, dietary fibers, lipids, phenols, organic acids, trace elements, phosphorylated sugars, nucleotides, and nucleic acids [167,227,228]. A typical hulled buckwheat seed contains 55%, 12%, and 4% of starch, proteins, and lipids, respectively, and around 20% of soluble carbohydrates and other components [229]. The seed contains approximately 10.9 g/100 g of dietary fiber; hence, it finds immense applications as a functional food ingredient (Li and Zhang, 2001). The fatty-acid profiling of buckwheat seeds, as given by various researchers, identified the presence of 18 fatty acids. Previous studies by Dorrell [230] concluded that palmitic, linoleic, linolenic, oleic, stearic, arachidic, behenic, and lignoceric acids contributed more than 93%, making them the major fatty acids. Similarly, previous studies by Gulpinar et al. [231] confirmed oleic acid to be the dominant unsaturated fatty acid in buckwheat seed oil, with palmitic acid as the main saturated fatty acid. The ethanolic extract of the seeds showed the prominence of rutin as an antioxidant and contributed to 20–21 mg/g of extract.

The presence of *L. plantarum*, *Bifidobacterium* sp. and *Bifidiobacterium lactis* in a buckwheat diet is an indication of its candidature as prebiotics [232]. A decrease in cholesterol levels in serum through its enhanced removal via the feces, sugar-absorption inhibition, and the improved growth of GIT microflora have been associated with the consumption of a buckwheat diet [1]. Patel and Goyal [1] revealed that the consumption of buckwheat resulted in an increase in lactic acid bacteria count compared with the control.

### 3.3. Pulses and Legumes

#### 3.3.1. Pulses

The major by-product of pulses is husk; a significant quantity of it is produced in India as they are the world’s largest producer of pulses [233]. Husk, the primary processing waste of pulse-milling units, contains pectic polysaccharides in the range of 1.4–5.3% [234]. These polysaccharides have been employed in the food industry as gelling and thickening agents for many years. Pulse-based waste could hence be considered an excellent source of plant-based polysaccharides, owing to the above property. Adding to this, the legume husk is also rich in dietary fibers (around 27–47%) of cellulose, hemicelluloses, lignin, and pectic substances and has immense importance in human nutrition [235]. Arulnathan et al. [20], in their study on the proximate composition of black gram husk from different regions of Tamil Nadu, reported a crude protein content of 15.70–22.56% and a crude fiber content of 14.12–24.09%. The hemicellulose and cellulose fractions of the husk were 9.99–11.88% and 25.32–28.34%, respectively.

#### 3.3.2. Legumes

The group known as legumes or legume seeds includes beans, fava beans, chickpeas, black gram, peas, lentils, cowpeas, lupins, lentils, and soybean [236]. The seeds are composed of starch as the major carbohydrate source and offer a low glycemic index, dietary fiber, oligosaccharides, and phenolics. The phenolic compound in legumes confers their antimicrobial and antioxidant potential, whereas the oligosaccharides modify the intestinal microbiota and aid the prebiotic potential [170]. Dietary fibers exist both in soluble and insoluble forms in legumes. The seed covering is rich in polysaccharides, whereas the cotyledon is of cellulose, hemicelluloses, and pectin. Studies focusing on the fermentation of legumes are employed widely, as fermentation is found to restrict anti-nutritional compounds, improve starch digestibility, and increase the antioxidant capacity of the seeds [170]. This has confirmed that prebiotic ingredients in the legumes support the growth of lactic acid bacteria [171]. The major amino acids present in legumes (tyrosine, phenylalanine, etc.) exhibit antioxidant potential as well. Studies have reported that the oligosaccharides in legumes sometimes act as anti-nutritional factors. However, it was concluded that they form part of the dietary fiber and exhibit prebiotic effects by stimulating the growth of Lactobacilli and Bifidobacteria and limiting the growth of harmful Enterobacteria [171,236]

### 3.4. Oil Seeds

#### Sesame Seeds

The sesame seed (*Sesamum indicum* L.) or benniseed is an oil seed that harbors approximately 40–60% of oil, 19–25% of protein, and 11.8% percent of dietary fiber [237]. This makes them an ideal candidate for food products focused on human nutrition. With the growing importance and the need to explore the bioactive potential of underutilized plant parts, various researchers have focused on sesame seeds. The presence of γ-tocopherol and sesamol in the sesame seeds and oils as antioxidants were reported by [238]. Further studies also revealed the presence of antioxidants, such as the bisepoxylignan analog and trans-ferulic acid, in sesame seeds [238]. The seeds are rich in furofuran lignans, which makes them a potential source of dietary lignans. The most prominant lignans in sesame seeds that are reported are the oil-soluble sesamin and sesamolin, the glucosides of sesaminol and sesamolinol [239,240,241]. Sesamin was reported to have antihypertensive, cholesterol-lowering, lipid-lowering, and anti-cancer activities. Sesame seeds displayed a prebiotic effect on the growth of the probiotic lactobacilli, *L. gasseri*, and *L. rhamnosus* in fermented milk [172].

## 4. Insights on Prebiotics

Nowadays, research findings regarding the partially, insufficiently exploited health application potentials of prebiotics are on the increase. One study was conducted by Everard et al. [242], where they investigated the role of prebiotics in obese mice. They posited that the consumption of prebiotics reduced the population of firmicutes, increased the level of glucose tolerance, decreased fat accumulation, and lowered oxidative stress and inflammation. The study established that the consumption of prebiotics induced the modulation of gut microbiota, improved glucose homeostasis, and can be utilized in diabetes mellitus therapy. It is, however, important for the researchers to conduct clinical studies, as well as to vary the different parameters that have been implicated in causing diabetes mellitus in the experimental rats. Patel and Goyal [1] reported that the prebiotic concept has been streamlined to fit the nutraceutical and pharmaceutical sphere; however, it is making exponential growth in the field of cosmeceuticals. A prebiotic mixture has been documented to have the potential to control microbial ecology in generating positive outcomes [1]. Recently, it has been hypothesized that prebiotics are effective in curing skin-related issues, such as inflammation and smelling odors. Bockmuhl [243] reported that prebiotic products have revealed an output of 91% success in an in vivo trial study using humans as subjects. This thereby affirmed that they can be utilized to significantly reduce the growth and treatment of acne caused by propionic bacteria. Similarly, Grüber et al. [244] x-rayed the effect of supplementation of infant formula with prebiotics on the occurrence of atopic dermatitis. They concluded that prebiotics containing the immune-active oligosaccharides could effectively prevent atopic dermatitis in infants, with a low risk of atopy-risk infants. These results could have been aided by the active growth stage of the infants and the breast milk intake, which can improve their natural immunity. Such experiments are good to be tested on older groups of people as a comparison with the present conclusions.

## 5. Conclusions

Wastes traditionally constitute a nuisance in the environment by causing air, water, and land pollution. The use of these seed wastes, which are naturally left to rot in the fields, will help in the synthesis of prebiotics targeted toward gut microflora. The insignificant side effects of prebiotics confirm their candidature as a food additive that can improve the microbial residents of the GIT. The role of prebiotics in animal husbandry and feed production has also been posited, consequently reducing the enormous usage of chemically synthesized antibiotics in animal agriculture. The wide range of using prebiotics in health promotion and maintenance cannot be over-emphasized; hence, research around prebiotics will continue to show an increase. The production of various prebiotics from seed wastes will boost not only the economy but also cause a reduction in environmental pollution. Concerted efforts should be made toward more experimental studies on several seed wastes to ascertain their potential as prebiotics.

## Figures and Tables

**Table 1:** Types of prebiotics.

Types	Examples	Functions	Reference
Fructans	Inulin FOS	Selective stimulation of LAB	[31,32]
Galactooligosaccharide	Lactose-based GOS Galactose-based GOS Raffinose family Oligosaccharide	Stimulation of Bifidobacteria and Lactobacilli	[24,27,31]
Starch- and Glucose-derived Oligosaccharides	Resistant starch Polydextrose	Butyrate production Stimulation of Bifidobacteria	[33,34]
Pectin Oligosaccharide			[24,35]
Miscellaneous	Cocoa-derived Polyphenols	Modulation of microbial diversity. Cell membrane integrity	[36]
	Glycoproteins		[37]
	Glycolipids		[37]

Key: FOS—fructo-oligosaccharides, GOS—galactooligosaccharides, POS—pectic oligosaccharides, LAB—lactic acid bacteria.

**Table 2:** Techniques used in the assessment of prebiotic efficacy.

Assessment Type	Techniques	Phases	Types of System	Type of Models	Conditions of Assessment						Samples to Be Evaluated	References
					Time (h)	pH	Temp (°C)	Detector	Salts	Enzymes		
Digestion of prebiotics	in vitro	Oral			5	7	37		NaCl	Alpha-amylase		[104]
		Gastric			2	2.5	37			Pepsin, Gastric Lipase		[105]
		Intestinal			2	6	37			Chymotrypsin, Trypsin, Colipase and Pancreatic Lipase		[106]
	In vivo			Animals	672		18–29				Fecal sample	[106,107]
				Human clinical	336						Undigested prebiotics	[106]
Fermentation of prebiotics	In vitro		Batch culture		24	6.5	37		Na_2_HPO_4_, NaH_2_PO_4_		Fecal sample	[22,108]
			Continuous culture	Single staged, multi-staged	16	6.8	37				Fecal sample	[109]
			Artificial gut	TIM	20	6	37				Fecal sample	[110]
				SHIME	24–72	6.5 7.0 7.5	37		Na_2_Co_3_		Fecal sample	[111]
	In vivo			Animals	672		22–29				Fecal sample	[112,113]
				Human Clinical							Fecal sample or Breathed air	[113]
Analysis of Prebiotics	SCFAs Analysis		HPLC		1	2.5	40	UV-Vis				[106,114]
			GC		0.5		250	FID				[106]
			HILIC		0.5		35	ELSD				[106,115]
			LC–ESI–MS.		0.5		35	DAD				[115,116]
	Gut Microbiota Enumeration	Culturing										[117]
		Molecular Methods		RT-PCR								[117]
				qPCR								[118]
				DGGE								[106]
				T-RFLP								[119]
				DNA Microarray								[106]
				16s rRNA								[106,120]
				Pyrosequencing								[106,120]

SCFAs—short-chain fatty acids, SHIME—simulator of the human intestinal microbial ecosystem, TIM—The Netherlands Organization for Applied Scientific Research intestinal model, FID—flame ionization detector, ELSD—evaporative light-scattering detector, DAD—diode array detector, T-RFLP—terminal restriction fragment length polymorphism, DGGE—denaturing gradient gel electrophoresis, RT-PCR—real-time polymerase chain reaction, qPCR—quantitative polymerase chain reaction, rRNA—ribosomal ribonucleic acid, HPLC—High-performance liquid chromatography, HILIC—Hydrophilic interaction liquid chromatography.

**Table 3:** Prebiotics and their health benefits in various industries.

Health Benefits	Functionality	Prebiotic Type	Industries	Product	Reference
Growth medium for starter culture	Reduction in generation time Reduction of linoleic acid content	Inulin FOS	Dairy	Cheese	[125,126]
GIT improvement	Constipation reduction Colitis prevention GIT microflora improvement	FOS Maltodextrin Mannooligosaccharide Arabinogalactans	Food Pharmaceutical	Infant formula	[1,3,7]
Anticancer and immune potentiator	Carcinogen reduction Primary bile acids to secondary bile acids conversion Promotion of T Helper 1 and regulatory T cells Regulation of IgE-mediated allergic responses	AXOS Epilactose GOS FOS, Pectins	Pharmaceutical	Supplemented diets Antibody Vaccines	[1,130,133]
Cardiovascular disease and obesity reduction	Satiety induction	FOS	Pharmaceutical	Supplements	[1,136]
Vaginal Health	Vagina microbiota restoration	Pectinate	Pharmaceutical	Bioadhesive	[1,138]
Agricultural feed	Mycotoxin reduction Growth enhancement Disease resistance	MNB XOS Inulin	Poultry Animal husbandry	Animal feeds Poultry feeds	[1,4,139]
Antibiotic Production	Inhibition of *E. coli*	Sorbitol Inulin Raffinose Lactulose	Pharmaceutical	Bacteriocin	[8,9]
Supplementations	Increased nutritional value Induction of LAB growth	AXOS	Dairy Baking	Milk Bread	[143,145]

Key: FOS—fructooligosaccharides, AXOS—arabinoxylanoligosaccharides, GOS—galactooligosaccharides, XOS—xylo-oligosaccharides, MNB—mannobiose, LAB—lactic acid bacteria.

**Table 4:** Potential applications of seed wastes as prebiotics.

Seed Groups	Seed Sub-Groups	Extraction Methods	Active Components	Uses	References
Fruit seeds	Date seed	Microbial fermentation	Dietary fibers	Cultivation of *Saccharomyces cerevisiae*	[153,154,155]
	Grape seeds	Aqueous	Proanthocyanidins	Antithrombotic Antitumor Anti-mutagenic	[156,157]
	Mango seeds	Ethanolic	Polyphenols	Modulation of gut microbiota	[158,159,160]
	Tamarind	Alcoholic	Monomers of glucose, galactose and xylose	Stimulation of LAB growth Anti-diabetic	[161,162]
Cereals	Rice	Aqueous	Dietary fibers	Satiety regulation Reduction in the glycemic index of food Prevention of diseases	[163,164]
	Brewer’s spent grains	Acid hydrolysis	Xylose	Increase in fat excretion Reduction of gallstones and plasma cholesterol	[165,166]
	Buckwheat	Ethanolic	Resistant starch	Decrease in cholesterol level Colon health improvement	[1,167]
	Coffee spent	Enzymatic hydrolysis	MOS	Stimulate growth of microbiota	[168,169]
Legumes and pulses	Beans	Acid and alkaline hydrolysis	Dietary fibers Phenolics	Support growth of LAB Antioxidant potential Anti-diabetic	[170,171]
Oil seeds	Sesame seeds	Organic solvent	Sesamin and Sesamolin	Anti-hypertensive Lowering of Cholesterols Anti-cancer Stimulation of LAB growth	[172,173]

MOS—mono-oligosaccharides, LAB—lactic acid bacteria.

## Data Availability

Not applicable.
